# Characterisation of SiPM Photon Emission in the Dark

**DOI:** 10.3390/s21175947

**Published:** 2021-09-04

**Authors:** Joseph Biagio McLaughlin, Giacomo Gallina, Fabrice Retière, Austin De St. Croix, Pietro Giampa, Mahsa Mahtab, Peter Margetak, Lars Martin, Nicolas Massacret, Jocelyn Monroe, Mayur Patel, Kurtis Raymond, Jolie Roiseux, Liang Xie, Guoqing Zhang

**Affiliations:** 1 TRIUMF, 4004 Wesbrook Mall, Vancouver, BC V6T 2A3, Canada; giacomo@triumf.ca (G.G.); fretiere@triumf.ca (F.R.); austindestecroix@gmail.com (A.D.S.C.); Pietro.Giampa@snolab.ca (P.G.); mmahtab@triumf.ca (M.M.); pmargetak@triumf.ca (P.M.); lmartin@triumf.ca (L.M.); nmassacret@triumf.ca (N.M.); mpatel@triumf.ca (M.P.); kraymond@triumf.ca (K.R.); jolieroiseux@gmail.com (J.R.); lxie@triumf.ca (L.X.); 2 Department of Physics, Royal Holloway, University of London, Egham TW20 0EX, UK; Jocelyn.Monroe@rhul.ac.uk; 3 Department of Physics, Simon Fraser University, 8888 University Drive, Burnaby, BC V5A 1S6, Canada; 4 Department of Physics, Engineering Physics & Astronomy, Queen’s University, 64 Bader Lane, Kingston, ON K7L 3N6, Canada; 5 SNOLAB, Lively, ON P3Y 1N2, Canada; 6 School of Science, Xi’an Polytechnic University, Xi’an 710048, China; zhangg_356@163.com

**Keywords:** Silicon Photomultipliers, Multi-Pixel Photon Counters, FBK VUV-HD3, HPK VUV4, spectroscopy, microscopy, dark noise, external cross-talk, nEXO, darkside

## Abstract

In this paper, we report on the photon emission of Silicon Photomultipliers (SiPMs) from avalanche pulses generated in dark conditions, with the main objective of better understanding the associated systematics for next-generation, large area, SiPM-based physics experiments. A new apparatus for spectral and imaging analysis was developed at TRIUMF and used to measure the light emitted by the two SiPMs considered as photo-sensor candidates for the nEXO neutrinoless double-beta decay experiment: one Fondazione Bruno Kessler (FBK) VUV-HD Low Field (LF) Low After Pulse (Low AP) (VUV-HD3) SiPM and one Hamamatsu Photonics K.K. (HPK) VUV4 Multi-Pixel Photon Counter (MPPC). Spectral measurements of their light emissions were taken with varying over-voltage in the wavelength range of 450–1020 nm. For the FBK VUV-HD3, at an over-voltage of 12.1±1.0 V, we measured a secondary photon yield (number of photons (γ) emitted per charge carrier ($e^{-}$)) of $\left(4.04\pm 0.02\right)\times 10^{-6}$ $\gamma /e^{-}$. The emission spectrum of the FBK VUV-HD3 contains an interference pattern consistent with thin-film interference. Additionally, emission microscopy images (EMMIs) of the FBK VUV-HD3 show a small number of highly localized regions with increased light intensity (hotspots) randomly distributed over the SiPM surface area. For the HPK VUV4 MPPC, at an over-voltage of 10.7±1.0 V, we measured a secondary photon yield of $\left(8.71\pm 0.04\right)\times 10^{-6}$ $\gamma /e^{-}$. In contrast to the FBK VUV-HD3, the emission spectra of the HPK VUV4 did not show an interference pattern—likely due to a thinner surface coating. The EMMIs of the HPK VUV4 also revealed a larger number of hotspots compared to the FBK VUV-HD3, especially in one of the corners of the device. The photon yield reported in this paper may be limited if compared with the one reported in previous studies due to the measurement wavelength range, which is only up to 1020 nm.

## 1. Introduction

Silicon photo-multipliers (SiPM)s have emerged as a compelling photo-sensor solution for detecting single photons in applications ranging from particle physics to medical imaging and ranging. SiPMs consist of an array of tightly packaged Single Photon Avalanche Diodes (SPADs) with quenching resistor operated above the breakdown voltage, $V_{\mathrm{bd}}$, to generate self-sustaining charge avalanches upon absorbing an incident photon. The excess voltage above breakdown is called *over-voltage*, and it is defined as $V_{\mathrm{ov}}\equiv \left(V-V_{\mathrm{bd}}\right)$, where *V* is the reverse bias voltage applied to the SiPM.

In contrast to the widely used Photomultiplier Tubes (PMTs), SiPMs are low-voltage powered, optimal for operation at cryogenic temperatures, and have low radioactivity [1]. Moreover, SiPMs have excellent Photon Detection Efficiency (PDE), not only in the visible and infrared wavelength range, but also for Vacuum Ultra-Violet (VUV) wavelengths [2]. For these reasons, SiPMs are the baseline solution in the DUNE experiment [3], aiming at precise neutrino oscillation measurements, the DarkSide-20k experiment searching for dark matter [4,5], and the nEXO neutrinoless double-beta decay search experiment [6].

The single-photon detection capabilities of SiPMs stems from its extremely high gain, since a single electron-hole pair can generate a charge avalanche on the order of $10^{5}$–$10^{7}$ electrons [7]. An unfortunate by-product of the avalanche generation process is the emission of secondary photons [8], which, in some works on SiPM characterisation, are referred to as *cross-talk* photons [9,10].

Secondary photons can be correlated with several factors: electric field, impurity concentrations, doping, geometry, etc., [11,12]. Even if an exhaustive list of production mechanisms is not known conclusively at present, avalanche emission in silicon appears to be due to a combination of (i) indirect interband transitions, (ii) intraband Bremsstrahlung processes and (iii) direct interband transitions [13,14,15]. Each of these mechanisms are responsible for light emission in different spectral regions, i.e., at certain wavelengths.

For example, avalanche emission below 2 eV appears dominated by indirect interband transitions, between 2 and 2.3 eV by intraband Bremsstrahlung and above 2.3 eV by direct interband [13,14,15]. The photon energy value for the transition from predominantly indirect interband to predominantly intraband Bremsstrahlung depends on the applied electrical field and material properties, while the transition from Bremsstrahlung to direct interband seems to occur at the same photon energy i.e., 2.3 eV [13].

Secondary photons in SiPMs are responsible for at least three processes: (i) internal cross-talk, (ii) external cross-talk and (iii) optically-induced afterpulsing. With internal cross-talk, we refer to secondary photons that subsequently trigger avalanches in neighbouring SPADs of the same SiPM without escaping from the SiPM itself. With external cross-talk, we instead refer to secondary photons that escape from the surface of one SPAD and potentially (i) reflect back into the SiPM at the surface coating interface and trigger avalanches in neighbouring SPADs [16], or (ii) transmit through the SiPM surface coating leaving the SiPM.

Finally, with optically-induced afterpulsing, we refer to secondary photons that trigger avalanches in the same SPAD that originated the primary secondary photon emission during the SPAD recharging time. Avalanches inside the same SiPM triggered by secondary photons can be simultaneous with the primary one (Direct Cross-Talk (DiCT)) or delayed by several ns (Delayed Cross-Talk (DeCT)) [17], and contribute to the total number of correlated avalanches per pulse produced by the SiPM [18]. The processes of DiCT and DeCT are extensively studied in literature with both measurements [19] and simulation [10]. We refer the reader to [19,20,21] for a detailed explanation of the different pulse-counting techniques used to discriminate these processes.

Secondary photon emission outside the SiPM that originally produced it can be problematic for large surface area, SiPM-based detectors since each SiPM can trigger other SiPMs in their vicinity, thus contributing to the detector background. For this reason it is important to study the SiPM secondary photon emission in order to quantify the systematic effects that hinder the overall detector performance.

This publication aims to study the emission spectra of the secondary photon emission outside the SiPM and its absolute secondary photon yield: number of photons (γ) emitted per charge carrier ($e^{-}$). Additionally, we investigate the uniformity of the light production over the entire SiPM surface area, identifying regions with heightened light emission intensity (hotspots), in agreement with other studies [22]. For this publication, we focused on two SiPMs, considered as photo-sensor candidates for the nEXO experiment: one Fondazione Bruno Kessler (FBK) VUVHD Low Field (LF) Low After Pulse (Low AP) SiPM (VUV-HD3) and one Hamamatsu Photonics (HPK) VUV4 Multi-Pixel Photon Counter (MPPC). Complete characterizations of the HPK VUV4 and FBK VUV-HD3 are reported in [6] and [18], respectively. Table 1 summarizes the SiPMs specifications relevant for this work.

The rest of this paper is organized as follows. In Section 2, we give a brief description of the setup used for measurements of the SiPM secondary photon emission, and we introduce its basic modes of operation. Overviews and analysis of our results for both imaging and spectroscopy of the biased SiPMs are provided in Section 3 and Section 4, respectively. Lastly, in Section 5, we provide some concluding remarks.

## 2. Triumf Characterization Setup

**Figure 1:**
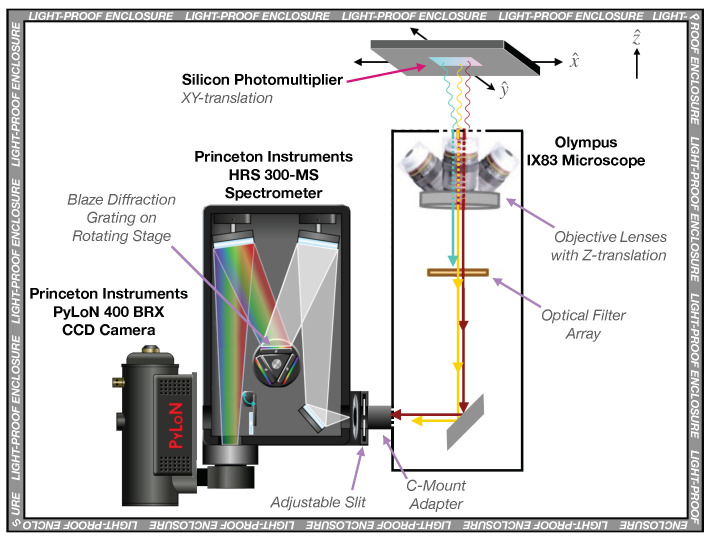
Schematic diagram of the TRIUMF apparatus used for SiPM imaging and spectroscopic measurements.

A new setup was developed at TRIUMF to characterize the light emitted by SiPMs, as illustrated in Figure 1. The setup comprises (i) an Olympus IX83 microscope, (ii) a Princeton Instruments (PI) HRS 300-MS Spectrometer and (iii) a PI PyLoN^®^ 400BR_eXcelon CCD camera. The SiPM is affixed to a translation stage above the microscope, with sub-micron motorized position adjustment in the XY-plane of Figure 1. The SiPM is biased by a Keithley 6487 Picoammeter, which is also used to monitor the SiPM current overtime. The entire apparatus is contained within a steel, light-proof enclosure, with all the components controlled externally. More precisely, the spectrometer is controlled by the PI LightField^®^ software, while the microscope is controlled by a combination of Olympus software and hardware.

The IX83 microscope incorporates (i) a filter cube array, used to insert filters in the light path in order to suppress second- or higher-order diffraction features in the measured spectra (depending on the wavelength range being probed); and (ii) an array of objective lenses. Table 2 summarizes the objective lenses installed in the TRIUMF setup along with their usage, as explained later in this section.

The PI spectrometer is attached to the microscope via a C-mount adapter and it is equipped with two blaze diffraction gratings: (i) a 300 lines/mm grating with peak efficiency at 300 nm and optimal transmission in the near ultraviolet (UV) wavelength range and (ii) a 150 lines/mm grating with peak efficiency at 800 nm and optimal transmission in the visible and near-infrared (NIR) wavelength range. These two gratings were chosen to maximize the spectrometer efficiency in the 450–1020 nm range.

Secondary photon emission in the UV and vacuum ultraviolet (VUV) is expected to be low, as shown in previously reported measurements [24]. Additionally, at room temperature, silicon can detect photons only up to 1107.6 nm due to its band-gap [25]. Photons with longer wavelengths may still be emitted, as shown in [24]; however, such photons cannot be detected by SiPMs. Moreover, the PI PyLoN^®^ 400BR_eXcelon CCD camera that is part of the PI spectrometer system is a silicon-based CCD that is not efficient beyond 1100 nm.

In addition to the camera and gratings, the spectrometer also has an adjustable input slit directly coupled to the C-mount adapter. The slit could also be removed entirely from the optical path, in order to capture emission microscopy images (EMMIs) of the SiPMs. More generally, for the measurements reported in Section 3 and Section 4, we used the setup in two basic modes of operation, which use different combinations of objective lenses, filters and gratings, summarized as follows.

1. Imaging mode

This measurement mode is used to record EMMIs of the biased SiPM in dark conditions, i.e., without external illumination. We used the PLCN4X-1-7 or LMPLFLN20X objectives (depending on the desired field of view) with no optical filters along the light path. Furthermore, the adjustable slit of the PI spectrometer was disengaged and the spectrometer grating (300 lines/mm) was set to its 0th-order.

2. Spectroscopy mode

The spectroscopy mode is used to measure the spectral components of the secondary photon emitted by the biased SiPM, also under dark conditions. To maximize the transmission of the PI spectrometer and the IX83 microscope in the wavelength range spanning 450–1020 nm, two combinations of gratings, filters, and microscope objectives were used. Within the 450–550 nm range, we used the LMPLFLN20X objective lens, no optical filters, and the 300 lines/mm PI grating centred on its first-order diffraction peak at 500 nm (this combination is hereafter called Visible Spectroscopy mode). Wavelengths between 550–1020 nm were measured using the LCPLN20XIR objective and the 150 lines/mm grating with its first-order peak centred at 800 nm.

Additionally, a 550 nm longpass filter was inserted along the IX83 light path to cut any second-order spectrometer features from wavelengths below 550 nm (this combination is hereafter called NIR Spectroscopy mode). For the measurements of the SiPM emission spectra reported in Section 4, the slit was set to a width of approximately 200 μm, corresponding to a wavelength width in Full Width at Half Maximum (FWHM) of 0.6 nm for the 300 lines/mm grating, and 1.4 nm for the 150 lines/mm grating.

The entire apparatus was calibrated in wavelength and intensity using the PI IntelliCal^®^ calibration system [26]. This system includes two light sources:

(i) A Hg and Ne-Ar line source for wavelength calibration with emission lines between 200 nm and 1000 nm.
(ii) A NIST traceable LED based light source for relative intensity calibration in the range 450–1020 nm

**Figure 2:**
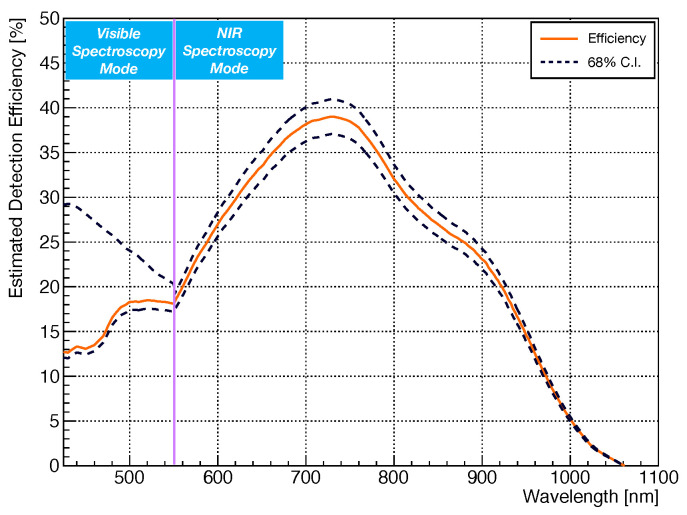
Estimated detection transmission efficiency of the TRIUMF apparatus as a function of the photon wavelength. The 68% Confidence Interval (C. I.) error bands account for systematic uncertainty in the lamp calibration. Below 550 nm, the error increases due to a disagreement between the observed IntelliCal^®^ LED based light source spectrum and its expected spectrum obtained combining the different hardware transmission specifications of the setup.

To perform these two calibrations, the SiPM was first substituted by the IntelliCal^®^ line source in order to calibrate the wavelength dependence of the PI spectrometer, and then by the IntelliCal^®^ intensity light source to assess the photon detection efficiency versus wavelength of the entire setup. Figure 2 reports the measured detection efficiency of the TRIUMF setup as a function of the wavelength.

The error bands account for the systematic uncertainty in the lamp calibration. Below 550 nm, the error increases for decreasing wavelength due to a disagreement between the observed IntelliCal^®^ LED based light source spectrum, measured with the CCD camera, and its expected spectrum obtained combining the different hardware transmission specifications of the setup. The discrepancy is not within the microscope. However, we could not determine if the discrepancy stemmed from miscalibration of the source or from a significantly lower transmission of the spectrometer + camera system in the visible wavelength range. As the light emission below 550 nm is small [24], the large error band was deemed acceptable.

## 3. Imaging of the Biased Sipm

**Figure 3:**
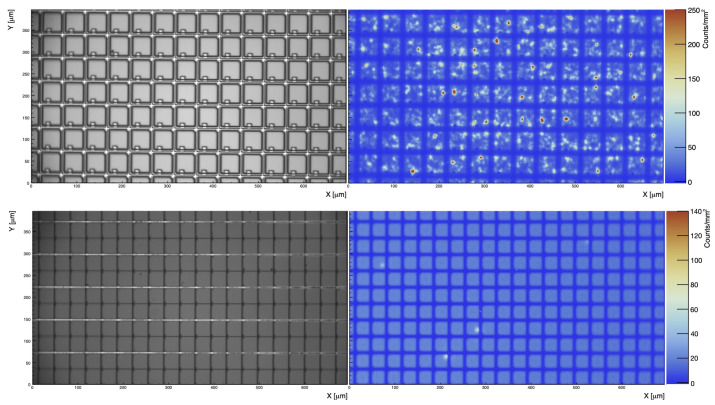
Top row: Emission microscopy image (EMMI) of the HPK VUV4 biased at 11.2±1.0 V of over voltage. Bottom row: EMMI of FBK VUV-HD3 biased at 13±1 V of over voltage. Both EMMIs were taken with the same camera exposure time and objective lens: LMPLFLN20X (20× magnification).

**Figure 4:**
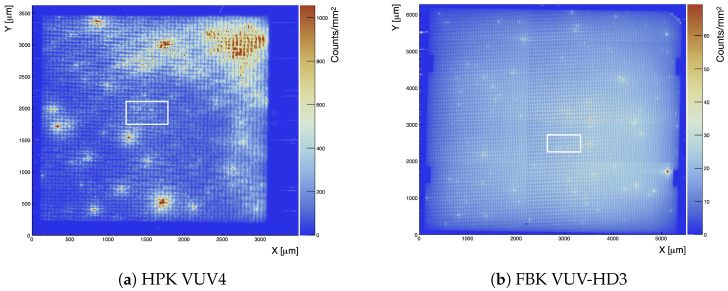
Composite EMMI of the HPK VUV4 and FBK VUV-HD3 SiPMs at 4× magnification and *V*_ov_ = 11.0 V and 13.0 V (±1.0 V), respectively. The regions enclosed in the white boxes are the areas where we zoomed to 20× magnification for spectral measurements. Both EMMI were taken with the same camera exposure time and objective lens: PLCN4X-1-7.

The imaging mode was used to record EMMIs of the biased SiPM as shown in Figure 3 and Figure 4. These EMMIs were used to compare the geometrical fill factors and topographical variations in photon emission for the HPK VUV4 MPPC and the FBK VUV-HD3 SiPM biased at 11.2±1.0 V and 13.0±1.0 V of over-voltage, respectively. At these two over-voltages, the current of the two SiPMs was roughly 2 mA. If we make the assumption that the SiPM current is entirely due to charge avalanches (the leakage current (i.e., not amplified current) could contribute to the total current realized by a SiPM [27]), and the photon emission is proportional to the total amount of charge generated by the SiPM, the two SiPM EMMIs in Figure 4, normalised to the same current level per unit area (The two SiPMs under study have different surface areas, and therefore Figure 3 and Figure 4 were scaled accordingly), can be used to compare the relative photon emission intensity and uniformity of the two SiPMs under investigation.

Since the number of photons emitted per charge carrier is rather low (on the order of $10^{-6}$ $\gamma /e^{-}$, see Section 4), and this paper focuses on SiPM photon emission driven by avalanche pulses generated in dark conditions, the high over-voltage is needed to generate a sufficiently large number of carriers in the SiPMs such that the light emitted by the SiPM was resulting in a reasonable signal to noise at the PI CCD camera. Figure 4 shows the entire surface of both SiPMs, combining several EMMIs at 4× magnification.

The *z*-scales in Figure 4a,b show that the HPK VUV4 SiPM tends to have brighter regions with enhanced light intensity (hotspots) compared to the FBK VUV-HD3, for which the hotspots appear more randomly distributed and within single SPADs. More generally the RMS of the light emission of the HPK MPPC is 3.3 times greater than that of the FBK SiPM, and behaves comparably to the one reported in [22] for KETEK PM3350T STD/MOD SiPM.

## 4. Spectroscopy of the Biased Sipm

The spectroscopy mode of the TRIUMF setup was used to measure the spectral shape of the secondary photon emitted for each biased SiPM, as introduced in Section 2. The emission spectra were recorded with the PI LightField^®^ software after calibration of the setup with the IntelliCal^®^ sources (Section 2). The net normalization from raw ADC Units (ADU)s recorded by the PI camera, $N_{\mathrm{ADU}}\left(\lambda \right)$, to the number of photons (γ) emitted per charge carrier, $N_{\gamma }\left(\lambda \right)$ [$\gamma /e^{-}$], at a given wavelength (λ) was obtained as follows

(1) $$N_{\gamma }\left(\lambda \right)\;=\;\frac{N_{\mathrm{ADU}}\left(\lambda \right)\;\eta_{\mathrm{ADU}}^{\gamma }\;q_{e}}{Q_{\mathrm{ET}}}\left(\frac{1}{\rho_{\mathrm{surf}}\;A\left(\lambda \right)\;\epsilon \left(\lambda \right)}\right),$$

where $\eta_{\mathrm{ADU}}^{\gamma }$ is the calibrated gain of the PI camera equal to 0.7γ/ADU (the camera gain is the independent from the wavelength since it refers to the gain applied by the preamplifier inside the camera after the exposure time has elapsed [28]); $q_{e}$ is the elementary electron charge; and $Q_{\mathrm{ET}}$ is the total charge that passed through the SiPM throughout the fixed exposure time $t_{\mathrm{ET}}$ defined as

(2) $$Q_{\mathrm{ET}}\;=\;\int_{0}^{t_{\mathrm{ET}}}i\left(t\right)dt$$

with i(t) SiPM current (The SiPM current was monitored during the entire exposure time with the Keithley 6487 Picoammeter (Section 2)). The parenthesis in Equation (1) represents a correction factor to account for photon losses in the TRIUMF setup. More precisely: $\rho_{\mathrm{surf}}$ represents the fraction of the SiPM light emitted within the field of view of the spectrometer slit (Section 4.1), ϵ(λ) is the TRIUMF setup detection transmission efficiency, as shown in Figure 2, and A(λ) is a photon acceptance correction factor that accounts for (i) the finite numerical aperture (NA) of the microscope objectives lenses and (ii) the reflection and absorption losses due to the SiPM surface coating and to the location of the avalanche region (Section 4.2).

During acquisition, the camera ADC readout rate was set to 50 kHz—the lowest speed available—to minimize the ADC readout noise. Table 3 summarizes for each measurement: the exposure time $t_{\mathrm{ET}}$, the over-voltage $V_{\mathrm{ov}}$, the average current 〈i(t)〉 and the total charge $Q_{\mathrm{ET}}$ that passed through the SiPM throughout the fixed exposure time, as defined by Equation (2).

### 4.1. Evaluation of the Correction Factor $\rho_{\mathrm{surf}}$

This section focuses on evaluating the correction factor $\rho_{\mathrm{surf}}$, used to account for the fraction of the SiPM light emitted within the field of view of the spectrometer slit.

**Figure 5:**
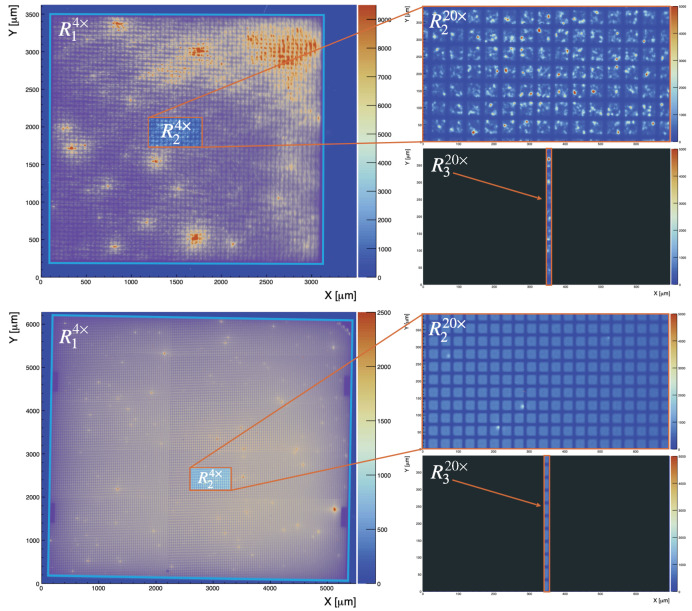
Pictorial representation of how the quantity $\rho_{\mathrm{surf}}$ is calculated for the HPK VUV4 MPPC (top) and FBK VUV-HD3 (bottom) SiPM combining the information of Figure 3 and Figure 4. $R_{k}$, with k = {1,2,3} are selected regions where the photon count is measured. See text for more details.

$\rho_{\mathrm{surf}}$ was computed recording EMMIs of the biased SiPM with and without the slit as follows:

(3) $$\rho_{\mathrm{surf}}\;=\;\left(\frac{\sum_{\begin{array}{c} i,j\subseteq R_{3} \end{array}}P_{i,j}^{20\times }}{\sum_{\begin{array}{c} i,j\subseteq R_{2} \end{array}}P_{i,j}^{20\times }}\right)\left(\frac{\sum_{\begin{array}{c} i,j\subseteq R_{2} \end{array}}P_{i,j}^{4\times }}{\sum_{\begin{array}{c} i,j\subseteq R_{1} \end{array}}P_{i,j}^{4\times }}\right).$$

where (i) $R_{k}$, with k = {1,2,3} are the regions highlighted in Figure 5 for the HPK VUV4 and for the FBK VUV-HD3, and (ii) $P_{i,j}^{M}$ are the number of photons counted in the i,jth pixel recorded by the CCD camera for an image at magnification *M*. Note as defining $\rho_{\mathrm{surf}}$ as done in Equation (3) not only accounts for the non-uniformity of the light emitted by the SiPM over its entire surface area, but also removes complications that may arise when comparing EMMIs taken with different magnifications.

The regions $R_{2}$ shown in Figure 5 are the same enclosed in white boxes of Figure 4. These regions are also the ones used in Section 4 to perform spectral measurements, after insertion of the slit. They were chosen for their centrality and for the absence of bright hotspots. The exact location of these regions is, however, not relevant since the spectra in Section 4 are always scaled using Equation (3) to account for the non uniformity of the light emitted by the entire SiPM. The two ratios of Equation (3) are in fact between counts of EMMIs taken with the same magnification, i.e., the same objective lens. The $\rho_{\mathrm{surf}}$ correction factors for the two SiPM tested are reported in Table 4.

### 4.2. Evaluation of the Correction Factor A(λ)

**Figure 6:**
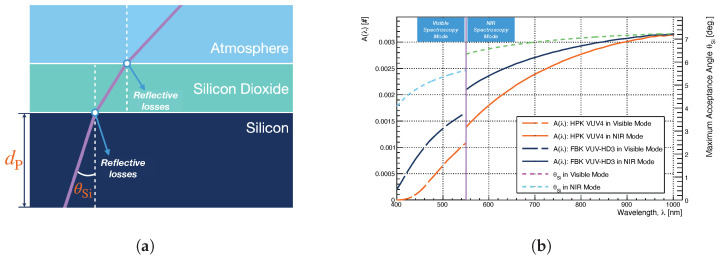
(**a**) Schematic representation of the SiPM surface coating structure used to compute the photon acceptance correction factor *A*(λ). *θ*_Si_ is the maximum angle for which photons emitted in the silicon can be detected by the microscope objective. *d*_P_ is the depth of the avalanche region. (**b**) The photon acceptance correction factor *A*(λ) (Equation (5)) and maximum acceptance angle (*θ*_Si_, Equation (7)) as a function of the wavelength for the two spectroscopy modes introduced in Section 2. The discontinuity in the *A*(λ) correction factor for the two SiPMs is due to the two different objective lenses (with different numerical aperture) used in the Visible (LMPLFLN20X) and NIR (LCPLN20XIR) spectroscopy measurement modes as shown in Section 2.

The photon acceptance correction factor A(λ) accounts for (i) the finite numerical aperture (NA) of the microscope objectives lenses, (ii) reflection and absorption losses due to the SiPM surface coating and to the location of the avalanche region. In what follows, we assume that the light emitted by SiPM avalanches is isotropic and not polarized. Additionally, the correction factor A(λ) is computed considering a SiPM surface coating structure constituted by a single layer of SiO${}_{2}$, as shown in Figure 6a.

This structure was provided by FBK and used in [29] for a study of the SiPM reflectivity. Hamamatsu did not disclose the HPK VUV4 surface coating structure, and therefore, due to the lack of more detailed information, we assume the HPK MPPC has the same coating structure as the FBK VUV-HD3 SiPM. The correction factor A(λ) was then computed neglecting interference and integrating over the solid angle [30] contained within the numerical aperture (NA) of the objective lens of the microscope as follows

(4)A(λ) = ∫02πdϕ∫02πdϕ∫0πsinθdθ∫0θSie−dPcosθμ(λ)(1−RSi,SiO2(λ,θ))(1−RSiO2,Atm(λ,θ′))sinθdθ(5) = 12∫0θSie−dPcosθμ(λ)(1−RSi,SiO2(λ,θ))(1−RSiO2,Atm(λ,θ′))sinθdθ,

where $e^{-\frac{d_{P}}{\cos\theta \;\mu (\lambda )}}$ (with μ(λ) attenuation length) is a correction factor to account for the self-absorption of the emitted photons in the silicon within a length $d_{P}$, as shown in Figure 6a. The avalanche region of each SiPM SPAD is in fact located at a certain depth ($d_{P}$) from the SPAD surface and the emitted photons need to travel a length equal to this depth before reaching the surface and escaping from it.

This self-absorption mechanism is significant for wavelengths below 450 nm due to the short attenuation lengths of UV photons in silicon [31], but it is negligible for longer wavelengths. The exact location of the avalanche region was not provided by FBK and HPK, however, with the model developed in [32], we can infer a lower limit to its depth. We will use the two depths reported in [32] to estimate $d_{P}$. More precisely, for the HPK VUV4, we used $d_{P}\;=\;0.8\pm 0.2\;\mu$m, while, for the FBK VUV-HD3 (that shares with the FBK VUV-HD1 studied in [32] the same surface coating and cell structure) $d_{P}\;=\;0.145\pm 0.01\;\mu$m. The wavelength dependent attenuation length was computed accordingly to the data reported in [31].

ϕ in Equation (5) is the azimuthal angle, $R_{{\mathrm{Si},\mathrm{SiO}}_{2}}$ is the reflectance at the silicon (Si)-silicon dioxide (${\mathrm{SiO}}_{2}$) interface, $R_{{\mathrm{SiO}}_{2},\mathrm{Atm}}$ is the reflectance at the silicon dioxide (${\mathrm{SiO}}_{2}$)-atmosphere (Atm) interface.

Both quantities were computed as reported in [30]. Finally θ is the emission angle of photons in the Silicon, and $\theta_{\mathrm{Si}}$ is the maximum angle for which photons emitted in the silicon can be detected by the microscope objective. This last quantity is reported in Figure 6b, and it is determined using the definition of NA and Snell’s law [30] as follows

(6) $$\begin{array}{cc} NA & \equiv n_{{\mathrm{SiO}}_{2}}\;\sin\theta_{{\mathrm{SiO}}_{2}}\;=\;n_{\mathrm{Si}}\;\sin\theta_{\mathrm{Si}} \end{array}$$

(7) $$\begin{array}{cc} ∴\;\theta_{\mathrm{Si}} & \;=\;\sin^{-1}\left(\frac{NA}{n_{\mathrm{Si}}}\right). \end{array}$$

with: $n_{i}$ and $\theta_{i}$ ($i\;=\;\{\mathrm{Si},{\mathrm{SiO}}_{2}\}$) as the refractive indices and photon angles (measured from the normal of the layer boundaries) of the Silicon (Si) and Silicon dioxide (${\mathrm{SiO}}_{2}$) medium. $\theta^{\prime }$ is similarly defined using Snell’s law as follows,

(8) $$\theta^{\prime }\;=\;\sin^{-1}\left(\frac{n_{\mathrm{Si}}\left(\lambda \right)}{n_{{\mathrm{SiO}}_{2}}\left(\lambda \right)}\sin\left(\theta \right)\right).$$

Equation (5) is solved numerically using the refractive index data for each wavelength reported in [33,34]. Figure 6b shows the correction factor A(λ) as a function of the wavelength. The discontinuity in the A(λ) correction factor for the two SiPMs is due to the two different objective lenses (with different numerical aperture) used in the Visible (LMPLFLN20X) and NIR (LCPLN20XIR) spectroscopy measurement modes, as shown in Section 2. The NIR spectroscopy mode has a higher A(λ) i.e., smaller correction factor, due to the higher objective lens NA.

### 4.3. Evaluation of the SiPM Photon Yields

**Figure 7:**
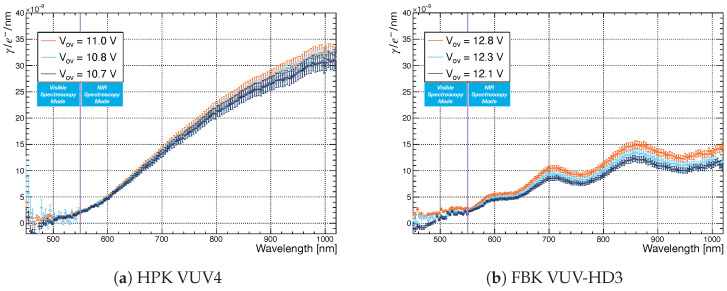
Spectra of the HPK VUV4 and FBK VUV-HD3 SiPMs as a function of the applied over-voltage.

Figure 7a,b report, for the two SiPM tested, the number of secondary photons emitted per charge carrier per nm $N_{\gamma }^{*}\left(\lambda \right)$, defined as

(9) $$N_{\gamma }^{*}\left(\lambda \right)\;=\;\frac{N_{\gamma }\left(\lambda \right)}{\Delta \lambda },$$

where Δλ represents the wavelength resolution, equal to 4 nm.

The uncertainties in Figure 7 were computed using a Monte Carlo simulation, assuming that (i) the photon emission of the SiPM follows a Poisson distribution, (ii) the systematic error of the efficiency correction is normally distributed and (iii) the detection probability of each CCD pixel of the PI camera follows a binomial distribution.

As shown in Section 2, the measured wavelength range was studied with two basic modes of operation that comprise different sets of objective lenses, filters and gratings to maximize the setup detection efficiency. These two modes are defined as Visible Spectroscopy ([450–550] nm) and NIR Spectroscopy ([550–1020] nm), depending on the wavelength range being studied (Section 2). After correction for the A(λ) factor, residual discontinuities in the spectra of Figure 7 were removed by measuring with the PI camera the wavelength range [550–640] nm with both the Visible and NIR Spectroscopy modes and averaging the CCD counts to obtain a smooth transition between the two modes.

The spectra in Figure 7 show that the secondary photon emission (i) is predominantly in the red and NIR, (ii) has a cutoff between 450 nm and 500 nm and (iii) increases with increasing wavelength and over-voltage. Since $N_{\gamma }^{*}\left(\lambda \right)$ is independent of the number of charge carriers flowing through the SiPM and the SiPM electric field increases with increasing over-voltage, the higher $N_{\gamma }^{*}\left(\lambda \right)$ for higher over voltage could be related to electric field dependent processes that contribute to the overall light production, as shown in [11,14,35,36].

The increasing $N_{\gamma }^{*}\left(\lambda \right)$ for increasing wavelength agrees with previously reported studies [13,14,15,24]. The FBK VUV-HD3, in particular, shows a clear signature of oscillations that are due to thin-film interference of light traversing the SiO${}_{2}$ surface coating. The HPK VUV4 does not show instead an interference pattern, probably due to its thinner coating. Geometry and surface coating can therefore contribute significantly to the final spectral shape.

The presence (FBK VUV-HD3) and absence (HPK VUV4) of an interference pattern was also measured in [29] during reflectivity measurements for the HPK VUV4 MPPC and the previous generation of FBK SiPMs: the FBK VUV-HD1, which shares with the FBK VUV-HD3 the same surface coating and cell structure.

The spectra in Figure 7 were measured up to 1020 nm. This is a consequence of the low Estimated Detection Efficiency (EDE) of the TRIUMF setup above 1020 nm, as shown in Figure 2. The EDE of the TRIUMF setup is in fact ∼2.6% at 1020 nm and <1% for wavelength above 1050 nm. Considering in fact a 200 μm spectrometer slit (Section 4), the signal to noise (S/N) for the longest exposure time (i.e., lowest over voltage) at the PI CCD camera was at 800 nm between 6 and 8 depending on the SiPM under study, and 1 at 1020 nm. Increasing the slit further would have increased the S/N but would have not significantly improved the capability of the TRIUMF setup to measure spectra above 1020 nm since the estimated detection efficiency of the system is low above 1020 nm.

Figure 7 can additionally be used to compute the total number of secondary photons emitted per charge carrier (i.e., the SiPM secondary photon yield) by integrating $N_{\gamma }^{*}\left(\lambda \right)$ over the measured emission spectrum as follows

(10) $$N_{\gamma }\;=\;\int_{450\mathrm{nm}}^{1020\mathrm{nm}}N_{\gamma }^{*}\left(\lambda \right)\;d\lambda$$

The results are reported in Table 5 and compared with the ones reported in [15,24] measured using a S10362-11-100U HPK MPPC and a photo-diode, respectively.

A quantitative comparison with the results reported in [24] is not possible since the author did not provide information on the average current that was flowing in the SiPM during their measurement. The $N_{\gamma }$ reported in this work is instead smaller if compared with the one reported in [15], which covers a similar reverse current range (Table 3). A possible explanation could be searched in the different spectral range covered by the two studies.

In [15], the authors measured up to 1087 nm, while we limited our analysis to 1020 nm, due to the limited efficiency of the TRIUMF setup above this wavelength value (Figure 2). According to [15], the $N_{\gamma }$ continue increasing for increasing wavelength, and therefore limiting the integral of Equation (10) up to 1020 nm could affect the estimation of the SiPM secondary photon yield resulting in a lower value if compared with the one reported in [15].

**Figure 8:**
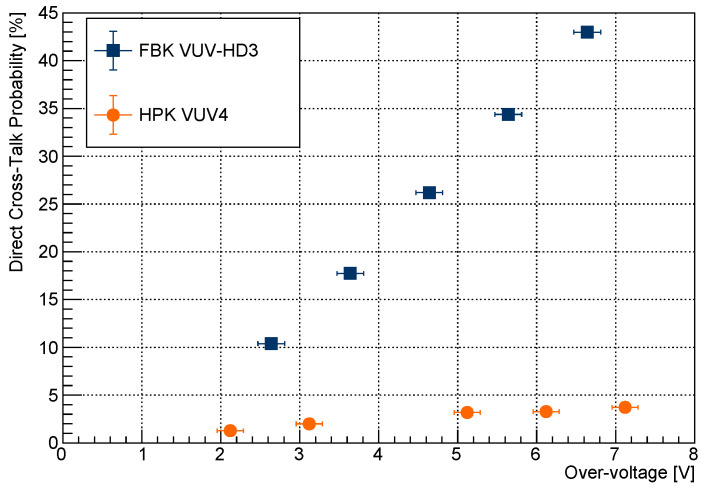
Direct cross-talk probability (DiCT) as a function of the applied over-voltage measured at 163 K for the two SiPMs tested in this work [6,18]. The DiCT probability is measured as the ratio between the number of prompt (or trigger) pulses with an integrated charge bigger than 1.5 Photo-electron Equivalent (PE) divided by the number of prompt pulses with an integrated charge bigger than 0.5 PE.

Overall, the number of secondary photons emitted per charge carrier by the HPK VUV4 is roughly a factor of two greater than that of the FBK VUV-HD3 SiPM. However the same is not true for internal cross-talk (i.e., DiCT, Section 1) since, as reported in Figure 8 the DiCT probability of the HPK VUV4 MPPCs is of the order of 3% at $V_{\mathrm{ov}}\;=\;4$ V, while the DiCT probability of the FBK VUV-HD3 is around 20% for the same $V_{\mathrm{ov}}$.

From this last point, we can deduce that (i) HPK trenches are extremely effective in suppressing internal cross-talk relative to FBK trenches [37]; and (ii) the reduction of the SiPM secondary photon emission does not necessarily follow the same design optimization loop compatible with the reduction of DiCT.

The DiCT probabilities reported in Figure 8 were measured at 163 K since the HPK VUV4 and FBK VUV-HD3 were tested in pulse counting mode in the context of the nEXO experiment.

The data reported in Figure 7 (and the results of Table 5) can be used as sampling distributions for a Monte Carlo simulation to estimate the probability of photon emission at a given wavelength per avalanche by each SiPM in the detector. Furthermore, paired with careful measurements of the SiPM Photon Detection Efficiency (PDE) [32] in the IR and NIR, this figure contains enough information to estimate the contribution of the SiPM secondary photon emission on the total background rate for any large-area SiPM-based detectors; this is crucial for experiments, such as nEXO and DarkSide-20k, where the SiPMs will likely be arranged facing each other.

We conclude this section stressing that, in this publication, we studied the SiPM secondary photon emission solely from dark noise-induced avalanches. The high over-voltage was then needed to ensure a reasonable signal to noise at the PI CCD camera. The number of secondary photons per charge carrier per nm reported in Figure 7 could, therefore, differ from the ones emitted at lower over voltages since, on average, SiPMs in pulse counting mode are operated at much smaller over-voltages than the ones reported in Table 5.

The data in Figure 7 were, however, normalized to the total generated charge in the SiPM and are, therefore, in principle, independent of the SiPM gain. Future analysis will focus on the SiPM secondary photon emission induced by laser-driven avalanches. This will allow not only the study of light propagation between neighbouring SPADs in the SiPMs but also to probe the emission spectrum at lower over-voltages.

## 5. Conclusions

SiPMs are arrays of SPADs separated by guard rings and other structures, such as trenches, to suppress optical cross-talk. Each SiPM SPAD is a reversely biased p-n junction that is operated above breakdown. In this configuration, a photo-generated carrier entering the depletion layer may trigger an avalanche. An unfortunate byproduct of the avalanche generation process is the emission of secondary photons, which, in some works on SiPM characterisation, are referred to as *cross-talk* photons.

Secondary photons can be correlated with several factors: electric field, impurity concentrations, doping, geometry, etc., and are responsible for at least three processes: (i) internal cross-talk, (ii) external cross-talk and (iii) optically-induced afterpulsing. With internal cross-talk, we refer to photons that subsequently trigger avalanches in neighbouring SPADs of the same SiPM without escaping from the SiPM itself.

Regarding external cross-talk, we instead refer to photons that escape from the surface of one SiPM SPAD and potentially (i) can be reflected back by the SiPM surface coating triggering avalanches in neighbouring SPADs of the same SiPM and (ii) can be transmitted through the SiPM surface coating leaving the SiPM itself. Finally, with optically-induced afterpulsing, we refer to secondary photons that trigger avalanches in the same SPAD that originated the primary secondary photon emission during the SPAD recharging time.

In this publication, we focused on the SiPM secondary photon emission outside the SiPM that can be potentially problematic for large-surface-area, SiPM-based detectors since SiPMs can trigger other SiPMs in their vicinity. For this reason, it is of primary importance to study the SiPM secondary photon emission in order to quantify the systematic effect that this mechanism can produce in the overall detector performance. For this publication, we focused on two SiPMs considered as candidate photo-sensors for the nEXO experiment: one FBK VUV-HD3 and one HPK VUV4 MPPC.

Spectral measurements of their light emission were taken with varying over-voltage in the wavelength range of 450–1020 nm. At an over voltage of 12.1±1.0 V we measure for the FBK VUV-HD3 a secondary photon yield of $\left(4.04\pm 0.02\right)\times 10^{-6}$ $\gamma /e^{-}$. Additionally, the light emitted by the FBK VUV-HD3 shows an interference pattern compatible with thin-film interference, and it presents a low amount of hotspots randomly distributed over the SiPM surface area. For the HPK VUV4 MPCC, at an over voltage of 10.7±1.0 V, we measured a secondary photon yield of $\left(8.71\pm 0.04\right)\times 10^{-6}$ $\gamma /e^{-}$.

Differently from the FBK VUV-HD3, the light emitted by the HPK VUV4 does not show an interference pattern, likely due to a thinner surface coating; however, it presents a large amounts of hotspots that tend to cluster on one of the corners of the device. The photon yield reported in this paper may be limited if compared with the one reported in previous studies due to the measurement wavelength range that is only up to 1020 nm.

## Figures and Tables

**Table 1:** Summary of the SiPM specification whose secondary photon emission is studied in this work [2,6]. The Fill Factor is defined as the ratio between the photon-sensitive area to the total area of the SiPM. The breakdown voltages are extracted from the SiPM I-V curves; defined as the voltage for which the first derivative with respect to the voltage of the SiPM current (in log space) is at the maximum [23].

Parameter	FBK VUV-HD3	HPK VUV4
Total Area	6 × 6 mm${}^{2}$	3 × 3 mm${}^{2}$
SiPM Fill Factor	80%	60%
SPAD pitch	35×35μm${}^{2}$	50×50μm${}^{2}$
Breakdown Voltage [298 K]	31 ± 1 V	52 ± 1 V

**Table 2:** List of Olympus objective lenses used in TRIUMF setup with their numerical apertures, magnifications and primary purposes.

Lens Model	Magnification	Numerical Aperture	Primary Use
PLCN4X-1-7	4×	0.1	Imaging
LMPLFLN20X	20×	0.4	Visible Spectroscopy
LCPLN20XIR	20×	0.45	NIR Spectroscopy

**Table 3:** Summary of the exposure time ($t_{\mathrm{ET}}$), over-voltage $V_{\mathrm{ov}}$, total charge $Q_{\mathrm{ET}}$ (as defined by Equation (2)) and average current 〈i(t)〉 during SiPM spectral measurements.

Exposure Time $t_{\mathrm{ET}}$		FBK VUV-HD3		HPK VUV4	
		Visible	NIR	Visible	NIR
	$V_{\mathrm{ov}}$ [V]	12.1 ± 1.0	12.1 ± 1.0	10.7 ± 1.0	10.7 ± 1.0
8 h 20 min	〈i(t)〉 [μA]	59.1 ± 1.2	66.2 ± 1.3	78.8 ± 0.6	33.3 ± 0.3
	$Q_{\mathrm{ET}}$ [C]	1.77 ± 0.12	1.99 ± 0.12	2.36 ± 0.12	1.00 ± 0.12
	$V_{\mathrm{ov}}$ [V]	12.4 ± 1.0	12.4 ± 1.0	10.8 ± 1.0	10.8 ± 1.0
4 h 45 min	〈i(t)〉 [μA]	87.1 ± 0.6	97.5 ± 1.0	46.4 ± 0.4	88.0 ± 0.6
	$Q_{\mathrm{ET}}$ [C]	1.49 ± 0.09	1.67 ± 0.09	0.80 ± 0.09	1.51 ± 0.09
	$V_{\mathrm{ov}}$ [V]	12.8 ± 1.0	12.8 ± 1.0	11 ± 1	11 ± 1
3 h 20 min	〈i(t)〉 [μA]	200.9 ± 1.7	165.5 ± 1.3	240.5 ± 1.3	187.8 ± 1.2
	$Q_{\mathrm{ET}}$ [C]	2.41 ± 0.08	1.99 ± 0.08	2.89 ± 0.08	2.25 ± 0.08

**Table 4:** Values of $\rho_{\mathrm{surf}}$ for the FBK VUV-HD3 and HPK VUV4 SiPMs.

	FBK VUV-HD3	HPK VUV4
$\rho_{\mathrm{surf}}$	$\left(1.72\pm 0.08\right)\times 10^{-4}$	$\left(2.40\pm 0.12\right)\times 10^{-4}$

**Table 5:** Photon yields (number of photons emitted per charge carrier) measured in the wavelength range [450–1020] nm for the FBK VUV-HD3 and HPK VUV4 SiPMs as a function of the applied over-voltage. The last line of the table represents the photon yields measured in [15,24], respectively.

FBK VUV-HD3		HPK VUV4	
$V_{\mathrm{ov}}$ [V]	Photon Yield [$\gamma /e^{-}$]	$V_{\mathrm{ov}}$ [V]	Photon Yield [$\gamma /e^{-}$]
12.1 ± 1.0	$\left(4.04\pm 0.02\right)\times 10^{-6}$	10.7 ± 1.0	$\left(8.71\pm 0.04\right)\times 10^{-6}$
12.4 ± 1.0	$\left(4.45\pm 0.02\right)\times 10^{-6}$	10.8 ± 1.0	$\left(8.98\pm 0.06\right)\times 10^{-6}$
12.8 ± 1.0	$\left(5.10\pm 0.02\right)\times 10^{-6}$	11.0 ± 1.0	$\left(9.24\pm 0.05\right)\times 10^{-6}$

Photon Yield in [24] (500–1117 nm):$\;1.2\times 10^{-5}$ $\gamma /e^{-}$; Photon Yield in [15] [0.5–4.5] mA (413–1087 nm):$\;2.9\times 10^{-5}$ $\gamma /e^{-}$.
